# Olfactory bulb proteome dynamics during the progression of sporadic Alzheimer's disease: identification of common and distinct olfactory targets across Alzheimer-related co-pathologies

**DOI:** 10.18632/oncotarget.6254

**Published:** 2015-10-28

**Authors:** María Victoria Zelaya, Estela Pérez-Valderrama, Xabier Martínez de Morentin, Teresa Tuñon, Isidro Ferrer, María Rosario Luquin, Joaquín Fernandez-Irigoyen, Enrique Santamaría

**Affiliations:** ^1^ Proteomics Unit, Clinical Neuroproteomics Group, Navarrabiomed, Fundación Miguel Servet, Proteored-ISCIII, Instituto de Investigación Sanitaria de Navarra (IdiSNA), Pamplona, Spain; ^2^ Pathological Anatomy Department, Navarra Hospital Complex, Instituto de Investigación Sanitaria de Navarra (IdiSNA), Pamplona, Spain; ^3^ Institut de Neuropatologia, IDIBELL-Hospital Universitari de Bellvitge, Universitat de Barcelona, L'Hospitalet de Llobregat, Spain, CIBERNED (Centro de Investigación Biomédica en Red de Enfermedades Neurodegenerativas), Spain; ^4^ Laboratory of Regenerative Therapy, Department of Neurology and Neuroscience Division, Centre for Applied Medical Research (CIMA), University of Navarra, Instituto de Investigación Sanitaria de Navarra (IdiSNA), Pamplona, Spain

**Keywords:** Alzheimer, neurodegeneration, olfactory bulb, proteomics, Gerotarget

## Abstract

Olfactory dysfunction is present in up to 90% of Alzheimer's disease (AD) patients. Although deposition of hyperphosphorylated tau and β-amyloid substrates are present in olfactory areas, the molecular mechanisms associated with decreased smell function are not completely understood. We have applied mass spectrometry-based quantitative proteomics to probe additional molecular disturbances in postmortem olfactory bulbs (OB) dissected from AD cases respect to neurologically intact controls (n=20, mean age 82.1 years). Relative proteome abundance measurements have revealed protein interaction networks progressively disturbed across AD stages suggesting an early imbalance in splicing factors, subsequent interrupted cycling of neurotransmitters, alteration in toxic and protective mechanisms of β-amyloid, and finally, a mitochondrial dysfunction together with disturbance in neuron-neuron adhesion. We also present novel molecular findings in the OB in an autopsy cohort composed by Lewy body disease (LBD), frontotemporal lobar degeneration (FTLD), mixed dementia, and progressive supranuclear palsy (PSP) cases (*n* = 41, mean age 79.7 years). Olfactory mediators deregulated during the progression of AD such as Visinin-like protein 1, RUFY3 protein, and Copine 6 were also differentially modulated in the OB in LBD, FTLD, and mixed dementia. Only Dipeptidyl aminopeptidase-like protein 6 showed a specific down-regulation in AD. However, no differences were observed in the olfactory expression of this protein panel in PSP subjects. This study demonstrates an olfactory progressive proteome modulation in AD, unveiling cross-disease similarities and differences especially for specific proteins involved in dendritic and axonic distributions that occur in the OB during the neurodegenerative process.

## INTRODUCTION

The olfactory bulb (OB) is the first site for the processing of olfactory information in the brain and its deregulation is associated with neurodegenerative disorders (NDs) [1, 2]. In many cases, the olfactory deficit is an early event of these diseases being considered as a premotor sign of neurodegeneration and consequently, a reliable premature marker of NDs [3]. It has been suggested that the potential origins of olfactory dysfunction may be the depositions and inclusions of Tau, β-amyloid, and α-synuclein proteins in the OB, olfactory tract, and olfactory cortex [2, 4, 5]. Moreover, the presence and severity of hyperphosphorylated Tau, β-amyloid, and α-synuclein pathology in the OB reflects the presence and severity of respective pathologies in other brain regions [1]. On the other hand, the reduction of the cholinergic centrifugal inputs to the OB and the increased number of the dopaminergic cells observed in the OB region [4, 6] have also been suggested as potential origins of smell loss.

Although olfactory involvement may also appear in healthy non-demented elderly subjects [7], olfactory dysfunction is present in up to 90% of AD patients [1]. Some studies suggest that olfactory dysfunction is an early event of AD, preceding the appearance of typical AD symptoms, such as memory loss, and dementia. Neuropathological studies have pointed out that olfactory centres are involved in early Braak stages [8], and OB pathology correlates with cortical AD pathology [2, 9, 10]. An OB atrophy and a significant reduction in olfactory performance have been detected in AD respect to control subjects using MRI and PET technologies [11, 12]. In view of these data, an in depth biochemical characterization of the neurodegeneration that occurs in the OB is mandatory as a first step for understanding early smell impairment in AD.

For several decades, neuroanatomical, volumetric, and histological approaches have been the gold standard techniques employed to characterize the OB functionality. However, little attention has been focused specifically on the molecular composition of the OB from the perspective of proteomics [13-15]. We consider that deciphering the progressive proteome-wide alterations that occurs in the OB derived from AD cases with different Braak staging, might help develop early diagnosis and identify potential therapeutic targets for AD.

In this study, we used mass-spectrometry based quantitative proteomics as a discovery platform in order to increase our knowledge about the patho-physiological mechanisms that are disturbed during the AD neurodegeneration in the OB. More than 200 differential proteins between controls and AD-related phenotype were detected, pinpointing specific pathways, protein interaction networks, and potential novel therapeutic targets that are modulated in specific Braak stages. Protein targets mainly involved in dendritic morphogenesis, neuronal injury and axonic distribution were evaluated in a cross-disease study, revealing common and distinct molecular perturbations between different Alzheimer-related co-pathologies, providing novel candidate proteins for a druggability assessment at the level of the OB.

## RESULTS

**Figure 1:**
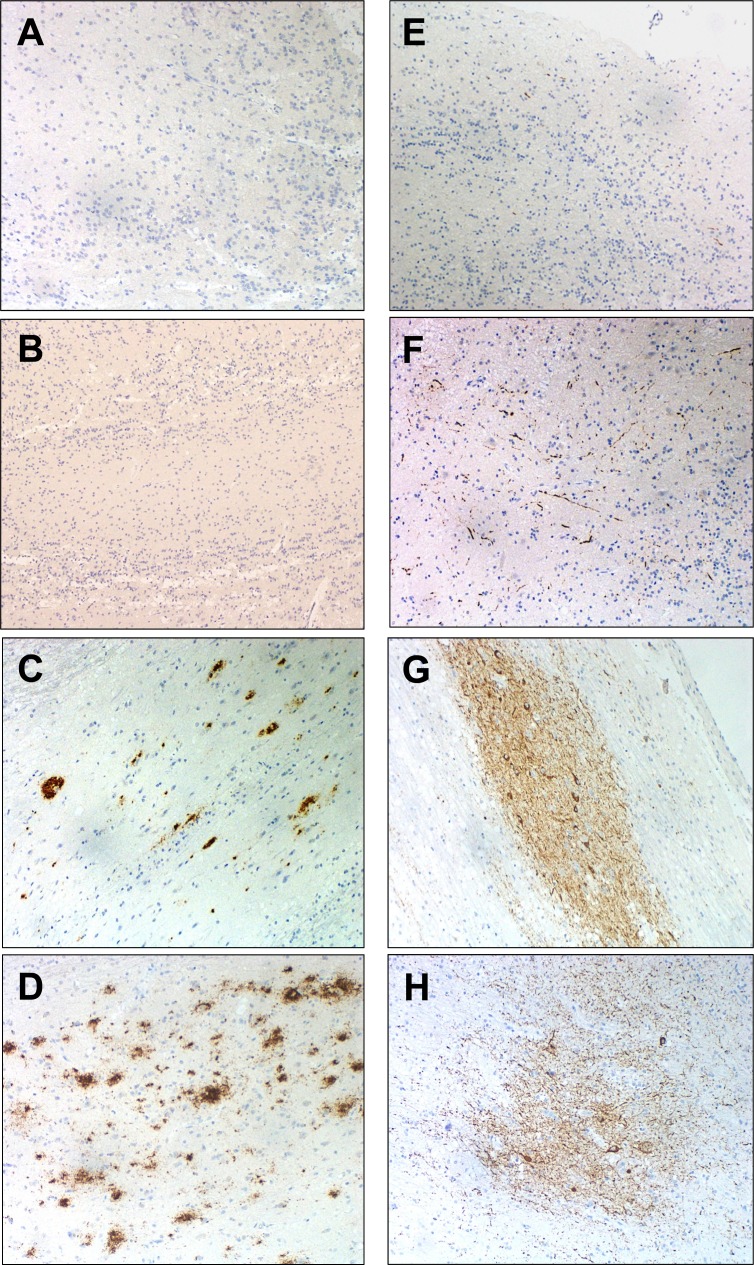
Representative immunohistochemical analysis of Beta-amyloid and p-Tau in OB Negative Beta-amyloid staining in control **A.** and initial stage **B.** moderate staining in intermediate stage **C.** and abundant deposit in form of diffuse and mature plaques in advanced stage **D.** Sparse neuropil p-Tau threats in OB glomerular layer in a control case **E.** p-Tau deposit in external layer of OB (initial stage) **F.** p-Tau threats and tangles in anterior olfactory nucleus (intermediate stage) **G.**, and abundant deposit of p-Tau in anterior olfactory nucleus (advanced stage) **H.** (all images are 20X).

In the present study, we have performed immunohistochemical analysis of β-amyloid and phosphorylated Tau in our OB sample collection derived from AD subjects (Table 1). The detection of β-amyloid was absent in nonpathological cases and increased along the progress of the disease. The morphology of the deposit was predominantly, in form of mature plaques in advanced stages, while in initial and intermediate stages, predominate the diffuse type of deposit. There was no specific anatomical localization of Aβ-protein in the OB (Figure 1). The detection of phospho-Tau protein was observed in all AD stages in form of neuropil threads and neurofibrillary tangles. The deposit was observed along the different layers of the OB. The intensity of phospho-Tau deposit was increased in the anterior olfactory nucleus (AON) of advanced stages of AD compared to initial stages and control cases where AON phospho-Tau staining was negative. In the OB of healthy patients there was only some isolated deposit of phospho-Tau protein (Figure 1). Although some variability in intensity and anatomical localization of protein aggregation were observed between different stages, our data allowed us to confirm the presence of neuropathological proteins in the OB from subjects with different Braak stages, reinforcing the involvement of the OB in pre-clinical stages of AD.

**Table 1:** Subjects included in the proteomic discovery phase

*Cases*			*Duration*	Brain	*PMI*	*Pathological diagnosis*	*IHQ*: β*A in OB*		*IHQ: TAU in OB*	
	*age*	*sex*	*(years)*	*weight (g)*	*(hours)*	*NIA-AA criteria*	*MP*	*DP*	*Tangles*	*neurites*
***Advanced stages***										
A1	77	F	16	946	4	AD (A2B3C3)	+	+++	+++	+++
A2	70	M	4	1104	2.5	AD (A3B3C3)	++	+++	++	+++
A3	89	M	13	1015	3	AD (A2B3C3)	+	+++	+++	+++
A4	86	M	8	973	2.5	AD (A3B3C3)	+	−	+	++
A5	93	M	3	1050	2.4	AD (A3B3C3)	+	+++	+++	+++
***intermediate stages***										
M1	85	M	12	1115	3.3	AD (A2B2C2)	−	+	+++	+++
M2	97	F	9	900	n.d	AD (A2B2C2)	n.d	n.d	n.d	n.d
M3	77	M	17	1103	1.5	AD (A2B2C1)	−	−	++	++
M4	99	F	n.d	1002	2.3	AD (A2B2C3)	n.d	n.d	n.d	n.d
M5	86	F	9	1000	3	AD (A2B2C2)	−	+	++	++
***Initial stages***										
I1	88	M	1	1400	3.45	AD (A2B1C2)	++	+	++	++
I2	85	F	8	1130	2	AD (A2B1C1)	−	−	+	+
I3	80	M	5	1090	3	AD (A2B1C1)	++	++	++	+++
I4	75	F	n.d	1125	6	AD (A1B1C1)	−	−	+	+
I5	72	F	n.d	810	4	AD (A1B1C1)	−	−	+	+
***Control***										
C1	66	M		1233	6.3	No protein deposit+vascular disease	n.d	n.d	n.d	n.d
C2	72	M		1407	9	Thal 1 Cerad 1 no tau deposit	−	−	−	+
C3	103	M		992	3	No protein deposit+vascular disease	−	−	−	+
C4	81	F		1176	3.3	PART (Braak I)+vascular disase	−	−	−	+
C5	61	M		1397	8	PART (Braak I)	−	−	+	−

The neuropathological assessment was performed according to Thal phases, CERAD score, NIA-AA guidelines and PART criteria [80-83]. βA immunopositivity was scored on a 4-tiered scale as: (−) negative, (+) 1-2 isolated βA depositions, (++) 3-4 βA depositions, and (+++) >4 βA depositions. Graduation of phospho-TAU deposit: (−) negative +: low; ++: intermediate; +++ high. PMI: post-mortem interval; n.d: not determined; MP: Mature plaques; DP: Diffuse plaques (See neuropathological study section).

### Large-scale identification of human olfactory bulb proteins by mass spectrometry

**Figure 2:**
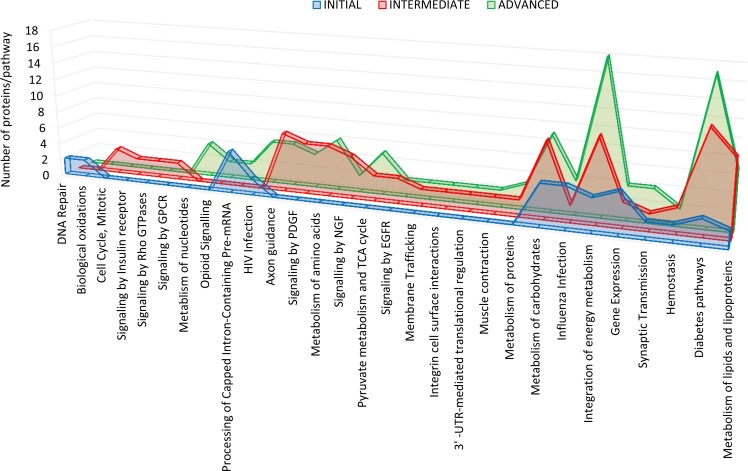
Differential olfactory proteome distributions across specific pathways Using Reactome database, differential OB proteome across AD stages was mapped into regulatory pathways (See Online Resource 6 for more details).

For shotgun proteomics experiments, we have used autopsy specimens of the right OB structure from AD cases and controls with no known neurological history (Table 1) with the final goal to obtain a profound insight into the protein content and protein function of the OB during the progression of AD. To screen the potential differences in OB protein expression profiles, OB specimens for each experimental group were separately subjected into isobaric tags (iTRAQ) coupled to 2D nano-liquid chromatography tandem mass spectrometry. Theoretically, pooling samples with a well characterized common pathologic phenotype, reduces the potential for aberrations in inter-sample, or biological variation. This reduction can improve the capacity to identify the most significant and consistent changes between stages. MS/MS data from OB structure were processed to identify peptides that gave rise to observed spectra, and proteins were inferred based on identiﬁed peptides. Using this workflow, we have generated an OB reference proteome map of 4,531 unique proteins identiﬁed with at least two peptides with ≥ 95% confidence (FDR lower than 1%). Complete lists of identifications and their corresponding scores are presented in Online Resources 2-3. To extract biological knowledge, the integrated OB proteome dataset was functionally categorized based on several pathway databases (online Resource 4). Although each bioinformatics platform produced diverse results, they commonly point out that tricarboxylic acid cycle, ubiquitin proteasome pathway and energy-releasing pathways are the general over-represented processes in OB as might be expected given the high metabolic demands of neurons (Online Resource 4). In order to gain a more detailed description of specific-neuronal pathways detected in the OB, subsequent analyses were performed to explore the proteome distribution across specific reactions using the PANTHER classification system [16]. Some statistically over-represented processes were directly relevant to synaptic vesicle trafficking, integrin signaling pathway, and histamine, dopamine, metabotropic glutamate and muscarinic acetylcholine receptor pathways (Online Resource 1-Figure 2, and Online Resource 4).

### OB proteome dynamics during the AD progression

**Figure 3:**
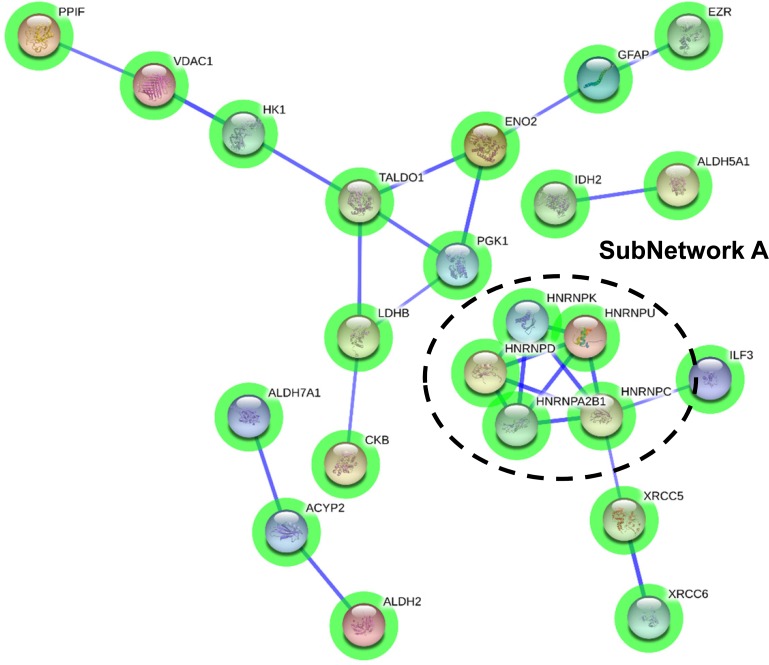
Protein interactome map for differentially expressed proteins in initial stages STRING analysis of known and predicted protein-protein interactions of gene-products differentially expressed in Braak stages I-II. STRING Version 9.1 and STRINGdb were used. Green circles: down-regulated proteins; red circles: up-regulated proteins.

To further understand the OB molecular background contributing to the progression of AD, we have performed a differential OB proteome analysis in order to detect early and stage-dependent molecular events underlying the progression of AD. As expected, we have detected a substantial heterogeneity within the same Braak staging. This may be due to unpredictable confounders such as clinical, environmental, behavioral and agonal factors (i.e medication, substance abuse and health status prior to death) [17]. Among the 4531 identified proteins, 231 proteins tend to be differentially expressed between controls and AD phenotypes (See Online Resource 1-Figure 3, and Online Resource 5 and 6). In order to gain a more detailed description of the molecular mechanisms involved in the OB during AD progression, subsequent analyses were performed to explore the differential olfactory proteome distributions across specific pathways (Online Resource 6). As shown in Figure 2, our results point out a stage-dependent deregulation of specific pathways. Protein clusters involved in DNA repair and biological oxidations were specifically mapped in initial stages while protein groups involved in cell cycle, and signaling by insulin receptor, Rho GTPases, and GPCR were exclusively detected in intermediate stages. In advanced stages, specific protein mediators of metabolism of nucleotides and opioid signaling were de-regulated (Online Resource 6). On the other hand, a de-regulation in protein clusters related to axon guidance, and signaling by PDGF, NGF, and EGFR appears throughout intermediate and advanced stages. Interestingly, a cumulative proteome disturbance is detected in energy-integrating and diabetes pathways during the AD progression at the level of the OB, suggesting novel experimental pieces of evidence that link diabetes with dementia [18].

**Figure 4:**
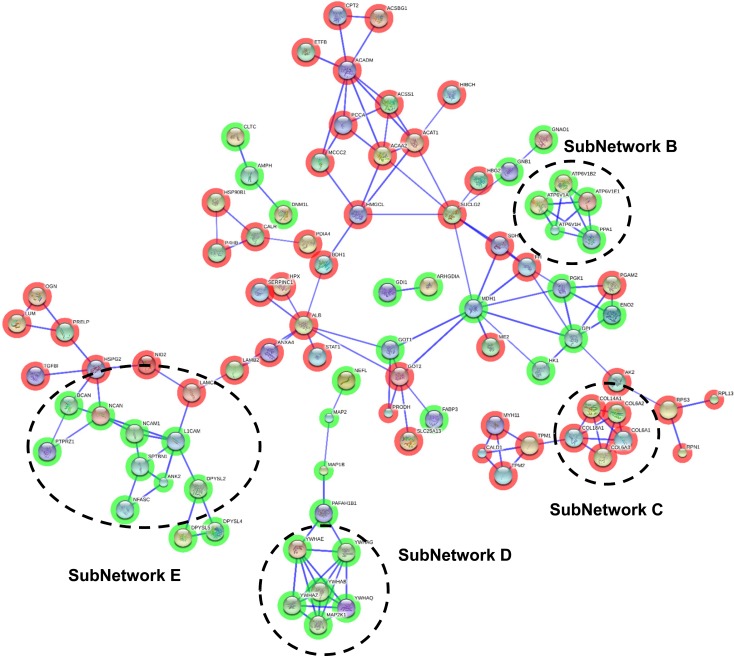
Protein interactome map for differentially expressed proteins in intermediate stages STRING analysis of known and predicted protein-protein interactions of gene-products differentially expressed in Braak stages III-IV. STRING Version 9.1 and STRINGdb were used. Green circles: down-regulated proteins; red circles: up-regulated proteins.

**Figure 5:**
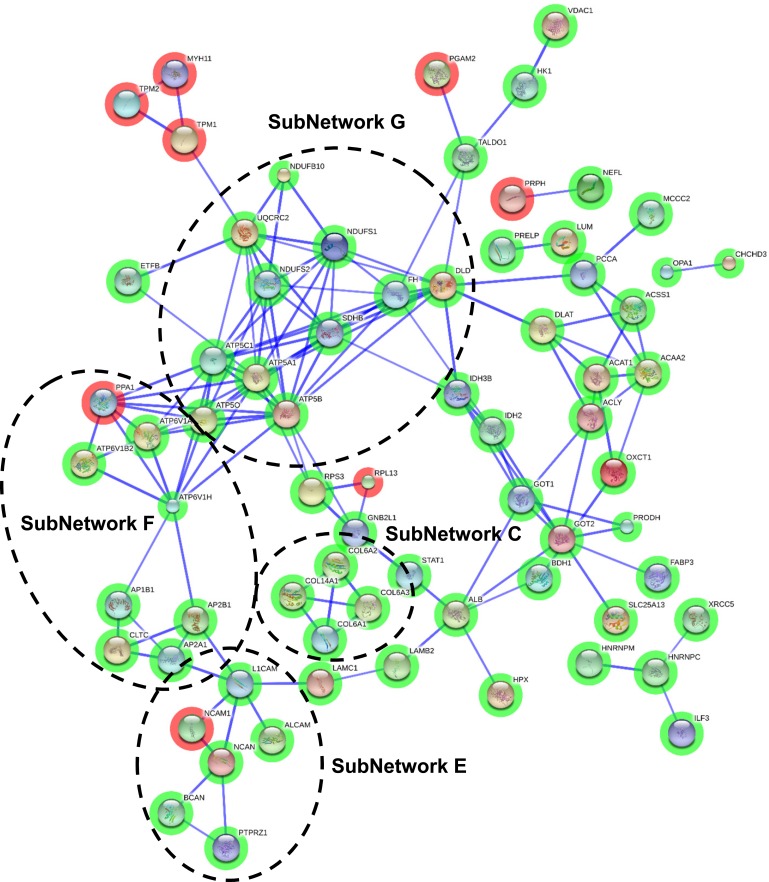
Protein interactome map for differentially expressed proteins in advanced stages STRING analysis of known and predicted protein-protein interactions of gene-products differentially expressed in Braak stages V-VI. STRING Version 9.1 and STRINGdb were used. Green circles: down-regulated proteins; red circles: up-regulated proteins.

To enhance the analytical outcome of proteomic experiments, we have performed proteome-scale interaction networks merging the olfactory proteins that tend to be de-regulated across stages of AD. Using STRING software, a protein interactome map has been constructed for each stage. In initial stages, the heterogeneous nuclear ribonucleoprotein system was compromised in OB (alteration in *HNRNPs*; subnetwork A) (Figure 3), whereas a deregulation in protein composition of V-type proton ATPase (subnetwork B), collagen (up-regulation of *COL6*, *COL14*, *COL18*; subnetwork C), 14-3-3 complexes (down-regulation of *YWHA* proteins; subnetwork D), and a disturbance in neuron-neuron adhesion (*L1CAM*, *NCAM1*, *ALCAM*, *NCAN*, *BCAN*; subnetwork E) appears in intermediate stages (Figure 4). In advanced stages, alterations in mitochondrial complex I (*NDUFs*), complex II (*SDHB*), complex III (*UQCRC2*), and complex V (*ATP5*) suggest an impaired mitochondrial function (subnetwork G) (Figure 5). Moreover, an imbalance in the neurotransmitter cycling (*ATP6Vs*, *AP-1*, *AP-2*; subnetwork F), and a disturbance in neuron-neuron adhesion and neurite growth (*L1CAM*, *NCAM1*, *ALCAM*, *NCAN*, *BCAN*; subnetwork E) also appear in advanced stages (Figure 5).

### Proteome-specific changes across AD staging

**Figure 6:**
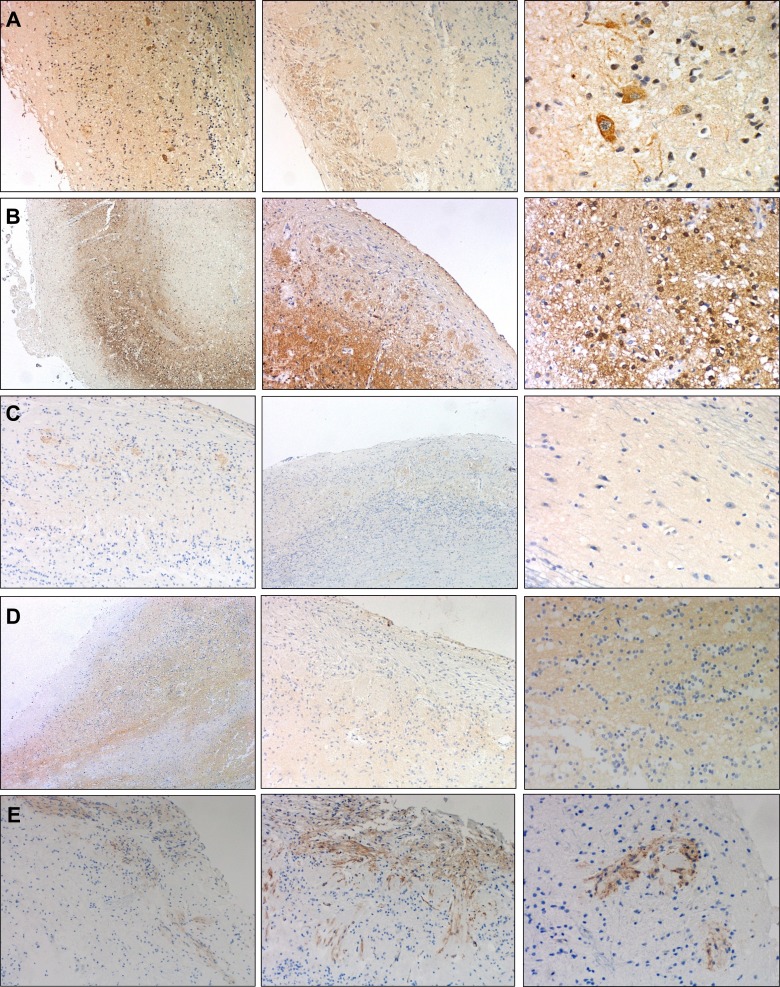
Immunohistochemical localization of AKAP12, RIPX, CPNE6, VILIP1, and DPP6 in human OB **A.** Representative immunohistochemical staining pattern of RIPX. Non-pathological OB (*left*), AD OB (advanced stage) (*middle*) (4-10X). Detail of RIPX expression in the cytoplasm and axons of mitral cells (40X) (*right*). **B.** Representative immunohistochemical staining pattern of CPNE6. Non-pathological OB (*left*), AD OB (initial stage) (*middle*) (4-10X). Detail of intense nuclear CPNE6 expression and neuropil deposit in granular cell layer (20X) (*right*). **C.** Representative immunohistochemical staining pattern of VILIP1. Non-pathological OB (*left*), AD OB (Advanced stage) (*middle*) (10X). Detail of diffuse expression of VILIP1 across neuropil in anterior olfactory nucleus (40X) (*right*). **D.** Representative immunohistochemical staining pattern of DPP6. Non-pathological OB (*left*), AD OB (advanced stages) (*middle*) (4-20X). Detail of DPP6 expression in granular cells. No evidence of nuclear and cytoplasm deposit (20X) (*right*). **E.** Representative immunohistochemical staining pattern of AKAP12. Non-pathological OB (*left*), AD OB (Advanced stage) (*middle*) (10X). Detail of AKAP12 expression in dendritic connections in glomerular cell layer (40X).

A total of 25 OB proteins were significantly differentially expressed proteins in specific stages (Table 2 and Online Resource 1-Figure 4). We compared these results with previous published data on protein differential abundance in AD generated by similar proteomic workflows. Out of 25 proteins, 10 were mentioned as differentially abundant in CSF, cortex, or hippocampus derived from AD subjects [19-24] (Table 2 and Online Resource 5). We investigate whether some differential olfactory proteins are locally modified at the first stages of AD-related pathology when morphological lesions are restricted to the entorhinal and transentorhinal cortices of neurofibrillary pathology (Braak I-II). For that, Copine-6 (CPNE6), Visinin-like protein 1 (VILIP1), Dipeptidyl aminopeptidase-like protein 6 (DPP6), and RUFY3 protein (RIPX) were further analyzed. To our knowledge, CPNE6, DPP6, and RIPX have not previously been associated with the pathogenesis and progression of AD. However, VILIP1 is a secretable calcium-mediated neuronal injury marker with potential diagnostic utility for early AD [25, 26]. Independent of its role as an auxiliary subunit of Kv4-mediated A-type K (+) channels [27], DPP6 participates in the dendritic morphogenesis during the neuronal development in the hippocampus [28]. RIPX is a poorly characterized protein involved in the formation of a single axon by developing neurons [29]. CPNE6 has been described as a protein regulator of axon maturation in the olfactory tract [30]. As shown in Figure 4 and 5, subnetwork C showed a de-regulation of Collagen VI chains in intermediate/advanced stages. Some studies have demonstrated that collagen VI protects neurons against β-amyloid toxicity [31]. Using FpClass tool as a data mining-based method for proteome-wide protein-protein interaction (PPI) predictions (http://dcv.uhnres.utoronto.ca/FPCLASS/) [32], we showed that AKAP12 protein (Src-suppressed protein kinase C substrate) is a putative interactor of Collagen VI chains. This protein increases tau phosphorylation and promotes β-amyloid toxicity in neuron-like cells [33]. First, we performed immunohistochemical analysis to localize these proteins in the OB region. As shown in Figure 6, RIPX tends to be expressed and preferentially distributed in the glomerular and mitral layers of the OB (Figure 6A). However, CPNE6 is highly expressed by granular and mitral cell layers. In some AD cases, we detected a focal deposit in glomerular structures (Figure 6B). VILIP1 staining is diffuse in glomerular layer presenting focal protein deposit in some areas (Figure 6C). On the other hand, DPP6 is basically detected across neuropil in granular and mitral cell layers, presenting in some cases negative staining in the glomerular layer (Figure 6D). In the case of AKAP12, positive staining was detected at dendritic connections in glomerular layer (Figure 6E). In some AD cases, AKAP12 expression was also detected in endothelial cells (Figure 6E).

**Table 2:** Top proteins significantly differentially expressed in specific AD stages

				Initial Stage (1)				Intermediate Stage (2)				Advanced Stage (3)					
Code	Name	CB / CA	p-Val CB/ CA	Stage 1 A/CA	p-Val 1 A/CA	Stage 1 B/CA	p-Val 1 B/CA	Stage 2 A/CA	p-Val 2 A/CA	Stage 2 B/CA	p-Val 2 B/CA	Stage 3 A/CA	p-Val 3 A/CA	Stage 3 B / CA	p-Val 3 B/CA	cortex	hippocampus
C9J2C0_HUMAN	Tubulin alpha-8 chain (Fragment)	0.87	0.05	0.53	0.00	0.71	0.01	0.93	0.23	0.46	0.00	0.98	0.86	0.86	0.25		
C9JFM0_HUMAN	Receptor-type tyrosine-protein phosphatase zeta	0.93	0.53	0.70	0.00	0.77	0.00	0.90	0.36	0.75	0.04	0.87	0.15	0.72	0.02		
DPP6_HUMAN	Isof. DPPX-S of Dipeptidyl aminopeptidase-like protein 6	0.92	0.36	0.80	0.04	0.72	0.00	0.72	0.01	0.71	0.00	0.73	0.00	0.55	0.00		
AT2B2_HUMAN	Isof. ZB Plasma membr. Ca2+-transporting ATPase 2	0.86	0.23	0.70	0.01	0.78	0.11	0.96	0.75	0.73	0.00	0.62	0.00	0.55	0.00		
VDAC1_HUMAN	Voltage-dependent anion-selective channel protein 1	0.94	0.11	0.74	0.00	0.91	0.02	1.02	0.58	0.85	0.24	0.75	0.00	0.47	0.01		
ILF3_HUMAN	Isoform 4 of Interleukin enhancer-binding factor 3	1.03	0.84	0.67	0.01	0.90	0.27	0.88	0.08	1.01	0.92	0.71	0.00	0.61	0.00		
B7ZLQ1_HUMAN	OPCML protein	0.81	0.17	0.91	0.55	0.76	0.01	0.88	0.31	0.79	0.07	0.60	0.04	0.47	0.00		
SYN2_HUMAN	Synapsin-2	0.90	0.30	1.25	0.00	0.90	0.49	0.93	0.60	0.61	0.00	0.69	0.01	0.54	0.01		
NFASC_HUMAN	Isoform 9 of Neurofascin	0.94	0.32	0.88	0.13	0.86	0.06	0.86	0.02	0.72	0.00	0.76	0.00	0.57	0.00		
BACH_HUMAN	Cytosolic acyl coenzyme A thioester hydrolase	0.94	0.42	0.81	0.01	0.86	0.04	0.95	0.64	0.65	0.00	0.72	0.00	0.57	0.00		
I3L0N3_HUMAN	Vesicle-fusing ATPase	0.96	0.48	0.99	0.88	0.90	0.09	0.97	0.53	0.65	0.00	0.75	0.00	0.59	0.00		
PACN1_HUMAN	PKC and casein kinase substrate in neurons protein 1	0.97	0.70	0.96	0.63	0.93	0.44	0.97	0.64	0.63	0.00	0.73	0.00	0.67	0.00		
HS12A_HUMAN	Heat shock 70 kDa protein 12A	0.91	0.21	1.22	0.02	0.85	0.00	0.97	0.67	0.71	0.00	0.77	0.00	0.61	0.01		
ATPO_HUMAN	ATP synthase subunit O, mitochondrial	0.98	0.81	0.82	0.03	0.91	0.22	0.96	0.49	1.08	0.22	0.64	0.00	0.56	0.00		
ATPG_HUMAN	ATP synthase subunit gamma, mitochondrial	0.91	0.62	0.79	0.01	0.82	0.02	0.91	0.28	1.02	0.86	0.67	0.00	0.57	0.01		
IDH3B_HUMAN	Isocitrate dehydrogenase [NAD] subunit beta, mit.	0.87	0.11	0.81	0.00	0.82	0.01	0.85	0.03	0.82	0.03	0.63	0.00	0.61	0.00		
NPTX1_HUMAN	Neuronal pentraxin-1	0.90	0.50	1.48	0.00	0.87	0.25	0.84	0.10	0.69	0.07	0.62	0.00	0.55	0.00		
BDH_HUMAN	D-beta-hydroxybutyrate dehydrogenase, mit.	0.98	0.89	1.01	0.95	0.75	0.04	0.90	0.49	2.03	0.00	0.71	0.04	0.57	0.01		
CALD1_HUMAN	Isoform 5 of Caldesmon	1.02	0.85	0.89	0.39	1.15	0.17	1.34	0.01	1.92	0.00	1.15	0.17	0.93	0.59		
PLSL_HUMAN	Plastin-2	1.22	0.30	1.11	0.17	0.99	0.94	1.42	0.00	1.54	0.00	1.25	0.07	0.77	0.16		
ADIRF_HUMAN	Adipogenesis regulatory factor	1.22	0.29	0.90	0.11	1.08	0.76	1.69	0.01	1.99	0.00	1.08	0.60	1.00	1.00		
KAD2_HUMAN	Adenylate kinase 2, mit.	1.19	0.06	1.03	0.89	1.16	0.28	1.38	0.00	1.91	0.00	1.13	0.44	1.01	0.97		
MYH11_HUMAN	Isoform 4 of Myosin-11	1.09	0.69	1.03	0.92	0.84	0.38	1.73	0.03	2.92	0.00	2.29	0.00	0.70	0.13		
H4_HUMAN	Histone H4	1.09	0.43	1.20	0.05	1.18	0.23	3.20	0.00	2.07	0.00	3.12	0.00	1.59	0.50		
K7EK07_HUMAN	Histone H3 (Fragment)	1.10	0.37	1.16	0.14	1.38	0.08	3.05	0.00	2.64	0.00	3.68	0.00	1.66	0.17		

CB, CA (control group A and B), Initial Stage (A and B), Intermediate Stage (A and B), and Advanced Stage (A and B) correspond to the iTRAQ experimental design (see Online Resource 1-figure 1). Ratio corresponds to the protein reporter ion intensity originating from control group B (tag114), Initial stage group A (tag115), Initial stage group B (tag116), Intermediate Stage group A (tag117), Intermediate Stage group B (tag118), Advanced Stage group A (tag119), Advanced Stage group B (Tag121) relative to control group A (tag113). Proteins were considered to show a significant downward (green) or upward (red) trend if their expression ratios were < 0.77 or >1.3 respectively. Proteins mentioned in previous reports as differentially abundant in cortex, or hippocampus derived from AD subjects are indicated.

**Figure 7:**
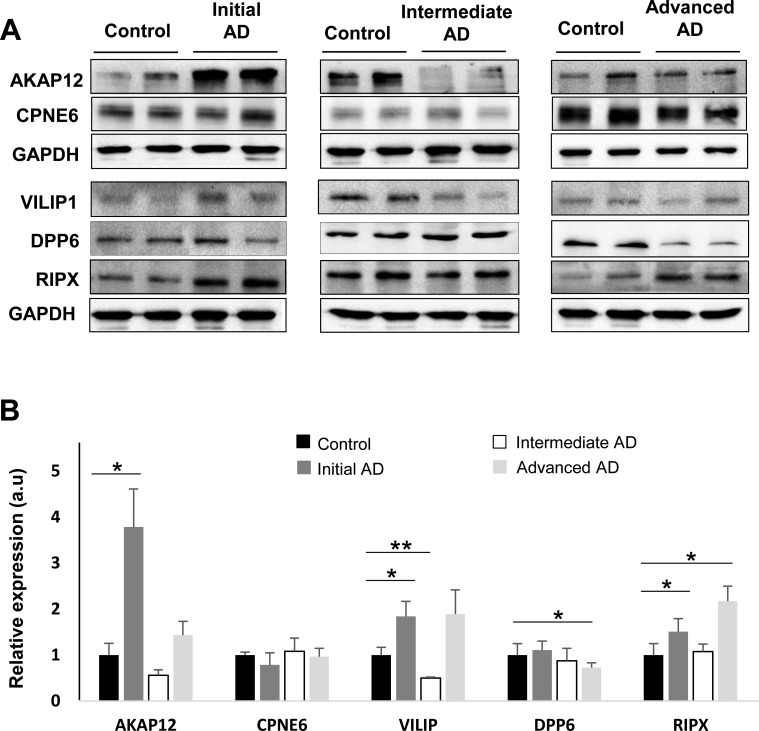
Olfactory bulb protein expression of *AKAP12, CPNE6, VILIP1, DPP6*, and *RIPX* across AD staging Protein expression were documented by Western blot analyses with antibodies against the respective proteins. Upper panel displays representative Western blot gels (*n* = 2/experimental group) to detect AKAP12, CPNE6, VILIP1, DPP6, and RIPX protein levels in the OB during the AD progression. Lower panel shows histograms of band densities. Data are presented as mean ± SEM from five independent OB samples per group. *P < 0.05 vs control group; **P < 0.01 vs control group.

Despite the fact that inter-individual variability generally is muted by pooling strategy, validation of individual cases using downstream assays is needed. With the aim to complement and partially validate the iTRAQ-based LC-MS/MS analysis, subsequent experiments were performed in order to check the steady-state levels of these proteins in individual cases by Western blotting. In accordance with our proteomic findings, olfactory VILIP1 was transiently up-regulated between early and intermediate stages respect to neurologically intact controls (Figure 7). In order to complement the tendency to DPP6 down-regulation in AD phenotypes detected by proteomics (Table 2), Western blot analysis were performed across AD stages. As shown in Figure 7, statistically significant differences were detected in olfactory DPP6 protein levels in advanced stages. These data suggest that the dendritic morphology and the number of functional synapses in OB neurons may be compromised in advanced stages of the disease [28]. As shown in Figure 7, RIPX was significantly over-expressed in the OB across early and advanced AD stages compare to controls, suggesting that RIPX overexpression may enhance the axon length, reducing the percentage of olfactory neurons with multiple axons in AD [29]. As shown in Figure 7, AKAP12 protein expression was significantly increased in early stages of AD compared to controls. However, AKAP12 tend to be down-regulated in intermediate stages. These findings reinforce the idea that transient increase in AKAP12 and collagen VI protein levels may be part of the protective mechanisms against β-amyloid in olfactory neurons of AD patients [31, 33]. Our proteomic analysis revealed a down-regulation of CPNE6 expression in a subset of patients across AD phenotypes (Supp table 5). In contrast, Western blot analysis revealed a tendency to down-regulation in early AD stages but no significant differences between AD phenotypes and controls was clearly detected (Figure 6). The divergence observed between mass spectrometry and Western blot data may be due to a possible biased quantification in our pooling strategy [34]. Overall, a consistent trend was observed with proteomics results for the selected proteins with the exception of CPNE6.

### OB protein expression of CPNE6, VILIP1, DPP6, and RIPX across Alzheimer-related co-pathologies

**Figure 8:**
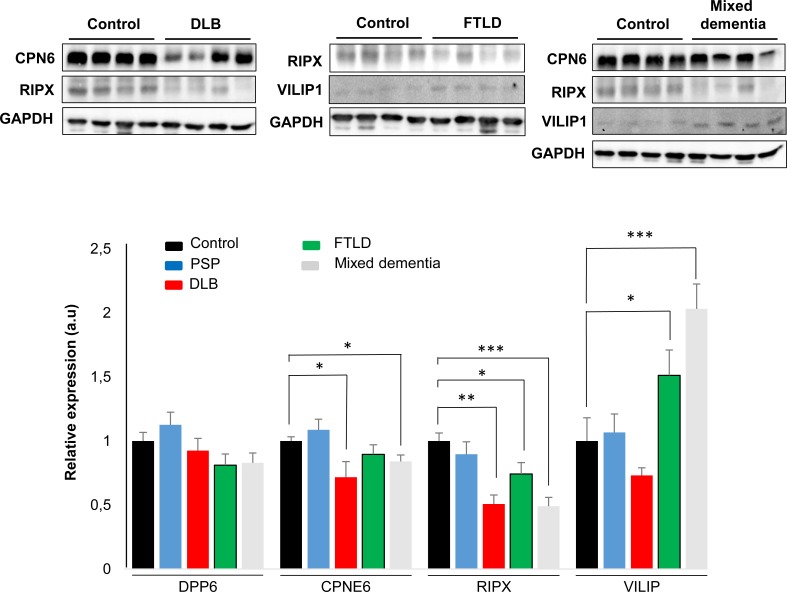
Olfactory expression of *CPNE6, VILIP1, DPP6*, and R*IPX* proteins across Alzheimer-related co-pathologies (*Upper panel*) Representative Western blot gels (n=4/clinical background) to detect CPNE6, VILIP1, and RIPX protein levels in the OB across different NDs. OB DPP6 protein levels remains unchanged across all neurodegenerative syndromes (data not shown). (*Lower panel*) Histograms of band densities derived from 41 independent OB samples. Data are presented as mean ± SEM from: Controls (*n* = 5 cases), PSP (*n* = 10 cases), LBD (*n* = 10 cases), FTLD (*n* = 6 cases), and mixed dementia (mix AD VD) (*n* = 10 cases). *P < 0.05 vs control group; ** *P* < 0.01 vs control group; ***P < 0.001 vs control group.

In contrast to the common separate investigation of NDs, targeted cross-disease studies comparing shared molecular relationships may give new insights into possible olfactory perturbations common for all or some NDs. In order to detect novel protein mediators shared by different Alzheimer-related co-pathologies at the level of the OB, we have evaluated the OB protein expression of CPNE6, VILIP1, DPP6, and RIPX by Western-blot across several AD-related diseases (*n* = 41 OB samples). We have included pathologies with common smell impairment like LBD, and FTLD [1], PSP where olfactory loss occurs to a lesser extent or is absent [1, 35, 36], and mixed dementia (Mix AD VD). Mixed dementia is a condition in which Alzheimer's disease and vascular dementia occur at the same time, and both separate disorders often display olfactory dysfunction [37, 38]. As shown in Figure 8, expression levels of our protein panel remained unchanged in the OB from PSP subjects respect to controls. Interestingly, although a tendency to DPP6 down-regulation is observed in FTLD and mixed dementia, a significant differential expression is not detected across NDs, suggesting that the alteration of olfactory protein expression of DPP6 tend to be specific for AD (Figure 8). In contrast, RIPX levels are significantly decreased in the OB from LBD, FTLD, and mixed dementia respect to protein levels detected in neurologically intact controls (Figure 8), whereas CPNE6 levels is differentially down-regulated in the OB from LBD and mixed dementia (Figure 8). On the other hand, olfactory VILIP1 expression is significantly up-regulated in FTLD, and mixed dementia. As equivalent olfactory deficits are observed across NDs, our data suggest that specific shared pathways including common pathological substrates are disturbed during the OB neurodegeneration in some Alzheimer-related co-pathologies.

## DISCUSSION

In view of the involvement of the olfactory dysfunction in AD, we consider that a quantitative knowledge of the olfactory neuroproteome across AD staging may help to understand the early smell impairment that occurs in this disease. The immunohistochemical study of β-amyloid and phospho-Tau allowed us to confirm the presence of neuropathological proteins in the OB of subjects with distinct stages of AD progression, confirming the involvement of OB in pre-clinical stages of the disease [1]. Although it is widely believed that OB perturbations are responsible for olfactory dysfunction in NDs [1], few studies have examined this area using high throughput molecular technologies. Although transcriptomic analysis have revealed multiple metabolic alterations in the OB of a rat AD model [39], the temporal progression of the disease in murine models does not correlate well with human AD [40]. Different neuroproteomic studies have been attempted to discover novel protein mediators associated with AD pathogenesis in brain areas differentially affected by the disease [41-44]. However, to our knowledge, this is the first study to characterize potential AD-associated molecular changes in the OB using MS-based quantitative proteomics. Basically, the discovery strategy used was based on differential labeling of peptides using isobaric tags prior to their separation and analysis by multidimensional LC coupled to MS [45], revealing new insights into the OB site-specific proteomic signature during the progression of AD. More than 4,500 proteins have been identified in human OB, being one of the most extensive proteomic characterization of a human brain area [46]. 231 proteins were significantly altered between some AD phenotypes and neurologically intact controls, where 11 potential biomarkers identified in the OB region (Serum albumin, 14-3-3 protein epsilon, Isoform 3 of Inter-alpha-trypsin inhibitor heavy chain H4, Antithrombin-III, Hemopexin, C4b-binding protein alpha chain, Gamma enolase, Phosphatidylethanolamine binding protein 1, Glial fibrillary acidic protein, Neuronal pentraxin-1, and Neurofascin) (see Online Resource 5) have already been proposed for their potential usefulness in AD diagnosis [23, 24, 47]. Interestingly, we have detected a down-regulation of specific HSP70 protein in OB from AD subjects (see Table 2). Intranasally-administered of recombinant human HSP70 in murine models of AD has demonstrated dramatic neuroprotective effects [48], suggesting that our experimental workflow is a useful approach to detect and identify practical therapeutic agents for AD treatment. Using data mining-based methods for proteome-scale PPIs predictions [32], we have generated the potential interactome for human APP (β-amyloid precursor protein) and Tau proteins (Online Resource 8), detecting some OB differentially expressed proteins during AD progression as potential APP and/or Tau interactors (Online Resource 1 Figure 5). Although these assumptions should be experimentally validated [49, 50], this predictive information may be useful to generate new working hypothesis to clarify the relationship between both neuropathologic substrates in AD.

One of the goals of the present study was to generate extensive data on the functional groups of proteins involved in the neurodegenerative process that occur in the OB during AD pathogenesis. For that, we have undertaken a system biology approach performing different molecular networks and protein profiling analysis [16, 51, 52] in order to identify biologically relevant pathways from large-scale OB proteome data. From a functional point of view, specific proteomic fingerprints are dynamically modulated in a stage-dependent manner throughout the OB. In initial stages, the heterogeneous nuclear ribonucleoprotein (hnRNP) protein complex was down-modulated (hnRNPA/B between others) (subnetwork A). It has been previously demonstrated that sporadic AD entorhinal cortices present a selective loss of hnRNP A/B splicing factors leading to the loss of synapses and dendrites [53]. Interestingly, hnRNP A1 up-regulation induces alternative splicing of the amyloid precursor protein (APP) mRNA, which is followed by reduced Aβ levels [54], suggesting that a fall of the hnRNP A/B protein levels may inversely exacerbate amyloid pathology in the OB.

In intermediate stages, we have detected an imbalance in the protein composition of V-type proton ATPase (subnetwork B). This multi-subunit protein drives the loading of neurotransmitters into synaptic vesicles [55], indicating that the cycling of neurotransmitters at the olfactory synapse is not totally efficient. Moreover, several Collagen VI chains (subnetwork C) and its potential interactor AKAP12 are de-regulated in intermediate stages, suggesting that modulation of its protein levels may be part of the protective mechanisms against β-amyloid in olfactory neurons of AD patients [31, 33]. 14-3-3 protein family plays a pivotal role in Tau phosphorylation [56], and also in oligomerization and aggregation [57]. Up-regulation of 14-3-3 members have been detected in cortex and choroid plexus of AD patients using proteomic technologies [22, 43]. In our case, olfactory 14-3-3 proteins (subnetwork D) tend to be down-regulated in olfactory neurons in intermediate stages. Albeit the brain areas analyzed are different, the apparent discrepancy between previous proteomic studies and our results may also be due to technological reasons. Previous reports have detected over-expression of 14-3-3 proteins using 2D-electrophoresis. This approach tend to detect proteoforms by resolving spots at different molecular weight and isoelectric point, hampering the analysis of total expression levels of each 14-3-3 isoform. However, it can be assumed that protein regulation determined by iTRAQ-based approach is more trustworthy regarding the overall expression change of a protein as they are not influenced by any isoform effects. In parallel, a disturbance in a subset of cell adhesion molecules (CAMs) and chondroitin sulfate proteoglycans appeared in intermediate stages, remaining altered in advanced stages (subnetwork E). In general, CAMs participate in neuron-neuron adhesion, and triggers signaling pathways to axon growth [58]. Specifically, L1CAM binds to β-amyloid reducing histopathological hallmarks of AD in mice [59], while NCAM1 participates in the maturation of the presynaptic endocytic machinery [60]. Furthermore, chondroitin sulfate proteoglycans such as neurocan (NCAN) and brevican (BCAN) are essential in the maintenance of hippocampal long-term potentiation [61, 62]. In view of these observations, the down-modulation of CAM-proteoglycan interactome may contribute to the β-amyloid-related synaptic toxicity impairment in the OB across intermediate-late phases of AD.

At the end stage of the disease, an interrupted synaptic transmission is largely reflected in the OB. There is a generalized inhibition in protein production of specific V-type proton ATPase subunits, Adaptor-related proteins (APs), clathrin heavy chain (CLTC) and CAM-proteoglycan complexes (subnetworks E and F), suggesting an immature form of synaptic vesicle recycling in the OB region [60]. Our proteome-scale interaction network analysis further revealed an impaired mitochondrial function, based on the down-regulation of subunits of mitochondrial respiratory chain complexes I, II, III and V (subnetwork G). This impairment is a common finding in human AD brains, and also in rodent, and cellular AD models [63, 64], where intracellular Aβ accumulation leads to a decrease electron transfer efficiency, reduce ATP production, and increase ROS production [65]. Overall, our data point out that functional protein interactomes and specific pathways are dynamically modulated across AD staging in the OB, emphasizing the potential impact of stage-dependent analysis using high-throughput proteome screenings.

Although the implementation of a pooling strategy reduce false-positive rates in proteomic experiments and partially control the clinical and pathophysiological heterogeneity [34], we further applied complementary approaches such as immunohistochemistry and Western blotting to analyze in detail the expression of Visinin-like protein 1 (VILIP1), Dipeptidyl aminopeptidase-like protein 6 (DPP6), Copine-6 (CPNE6) and RUFY3 protein (RIPX) in the OB. Both VILIP1 and RIPX are up-regulated at the first stages of AD-related pathology, when morphological lesions are restricted to the entorhinal and transentorhinal cortices. Interestingly, both proteins are also maintained in higher levels in advanced stages of AD. VILIP1 is a neuronal calcium sensor which CSF concentration is usually elevated in AD patients compared to control and LBD subjects [25, 26, 66], and predicts rates of future cognitive decline in early AD [67]. However, the increment in olfactory VILIP1 levels is transient, falling in AD subjects with intermediate stages in accordance with previous data obtained from cerebral cortex of AD patients [68]. It has been proposed that the down-regulation of VILIP1 may attenuate neuronal signaling pathways regulating the neuroplasticity process, contributing to cognitive decline in initial stages of AD [69]. In contrast, OB VILIP1 levels were increased in FTLD and mixed dementia subjects in our cross-disease study. Our data suggest that the complex pattern of up- and down-regulation of VILIP1 needs to be studied thoroughly in each pathology in order to decipher the specific signaling routes involved in VILIP1-dependent phosphorylation of Tau [70] and the role of VILIP1 in β-amyloid-induced calcium overload [71]. Interestingly, calcium sensors also modulate A-type K(+) channels, controlling responses to excitatory synaptic inputs [72]. DPP6 is an auxiliary subunit of Kv4-mediated A-type K(+) channels [27, 73], which deficiency induces a decrease in A-type current together with an independent reduction in the number of functional excitatory synapses, affecting the excitatory synaptic function and dendritic branch complexity in murine hippocampus [28, 74]. Our data also indicate a specific late reduction in the OB levels of DPP6 in AD, suggesting a potential contribution of DPP6 in the aberrant synapse stability at the end stage of AD-pathology. On the other hand, CPNE6 protein levels were only altered in the OB derived from LBD and mixed dementia (mix AD VD) indicating that the axon maturation process may be differentially compromised in the olfactory tract across different NDs [30]. RIPX is a poorly characterized protein involved in cytoskeletal dynamics in growth cones [75] to control the neuronal axon elongation [29]. We have observed a reversed olfactory pattern between mixed dementia (Mix AD VD) respect to the protein profile observed in AD, indicating that RIPX may be considered a common protein mediator that plays specific roles in axon guidance across different neuropathological backgrounds, compromising the regulation of cell polarity and membrane trafficking in olfactory neurons [76]. Transcriptional and translational events may explain this difference observed in RIPX protein levels. A possible explanation is that the vascular damage may induce an increment in the RIPX degradation rate at mRNA and/or proteins levels at the level of the OB in mix AD VD. However, we have to take into account that the activation/inhibition of the transcription factor machinery that regulate the transcription of RUFY3 gene may also be compromised (as a consequence of the vascular damage), leading to a decrease in RUFY3 mRNA and protein levels. However, additional validation studies should be conducted employing large cohorts to verify the protein expression changes observed in our sample set.

Although our study has uncovered many intricacies in OB protein homeostasis during AD progression, there are potential limitations of our study that warrant discussion. We have analysed dissected areas that contained multiple cell types [77], thus diluting the proteomic contribution of each specific cell type. Furthermore, the analysis of the OB is not sufficient to investigate the full magnitude of proteome modulation in AD across the olfactory system. Taking into account that neuropathologic substrates also tend to deposit in olfactory tract and olfactory cortex, additional proteomic studies targeted to these olfactory areas will be necessary to complement the proteomic data specifically derived from the OB.

## CONCLUSIONS

In summary, we have used a differential proteome-wide approach revealing stage-dependent molecular alterations in the OB during the AD progression. The differential proteomes lie in an imbalance in splicing factors, interrupted cycling of neurotransmitters, alteration in toxic/protective mechanisms of β-amyloid, mitochondria-mediated neurodegeneration, and a disturbance in neuron-neuron adhesion. Thus, our findings provide basic information for understanding the implication of the OB in the pathophysiology of AD, identifying protein mediators that may be used as potential therapeutic agents or even explored in biofluids as candidate biomarkers for AD diagnosis and evolution. In addition, our study revealed changes in the OB levels of specific proteins that had not previously been implied in neurological pathogenesis, suggesting that NDs that have markedly different clinical and pathological features present disruption of shared pathways at the level of the OB.

## MATERIALS AND METHODS

### Sample collection

According to the Spanish Law 14/2007 of Biomedical Research, inform written consent form of the Neurological Tissue Bank of Navarra Health Service was obtained for research purposes from relatives of patients included in this study. Fifteen AD cases were distributed into different groups according to specific consensus diagnostic criteria [78-80]: low, intermediate, and high neuropathological changes (*n* = 5/group) (Table 1). Five cases from elderly subjects with no history or histological findings of any neurological disease were used as a control group (Table 1). All brains considered in the discovery phase had a post-mortem interval (PMI) lower than 10 hours (Table 1). For specificity analysis, different NDs were considered: Progressive supranuclear palsy (PSP) (*n* = 10 cases; 5F/5M; median age: 74 years), Lewy body disease (LBD) (*n* = 10 cases; 6F/4M; median age: 80 years), frontotemporal lobar degeneration (FTLD) (*n* = 6; 3F/3M; median age: 81 years), mixed dementia (mix AD VD) (*n* = 10 cases; 5F/5M; median age: 85 years), and controls (*n* = 5; 2F/3M; median age: 79 years). 80% of the OB samples included in this phase had a PMI lower than 10 hours (See Online Resource 7).

### Neuropathological study

One hemisphere (usually the left) with the corresponding OB was fixed in 10% formalin for morphological studies. After fixation (21-25 days), representative brain areas from cortical and subcortical areas, brainstem, cerebellum and spinal cord were taken and embedded in paraffin in order to make a neuropathological diagnosis. Neuropathological assessment was performed according to standardized neuropathological scoring/grading systems, including Thal phases of Beta-amyloid deposition, Braak staging of neurofibrillary lesions, Consortium to Established a Registry for Alzheimer's Disease, National Institute on Aging-Alzheimer's Association (NIA-AA) guidelines, primary age-related tauopathy (PART) criteria, McKeith criteria for Lewy body disease, Mackenzie criteria for FTLD pathology, NINDS-AIREN criteria for vascular dementia, and NINDS criteria for PSP [78-87]. OB beta-amyloid and Phos-Tau immunostaining were exclusively performed in AD and control cases. The OBs were embedded in paraffin and 4-μm-thick sagittal sections were stained with hematoxylin-eosin and 3-μm-thick sagittal sections were processed for immunohistochemical analysis. Formalin-fixed sections (3-5 μm-thick) were mounted on slides and deparaffinized. After conducting a routine antigen retrieval protocol, tissue sections were immunohistochemically labelled overnight with a mouse monoclonal antibody anti-human PHF-TAU (clone AT-8, Innogenetics) and a mouse monoclonal (S6F/3D) anti Beta-amyloid (Leica). The reaction product was visualized using an automated slide immunostainer (Leica Bond Max) with Bond Polymer Refine Detection (Leica Biosystems Newcastle Ltd). Analysis of OB for specific protein deposits aggregates was carried out in a light microscope (Olympus BX51) blinded to pathological diagnosis. A semiquantitative assessment was performed according to Kovacs T *et al.* [88]. We consider compact deposit with central core as mature plaques and granular or fibrillar deposit as diffuse plaques. Moreover, Aβ immunopositivity was scored on a 4-tiered scale as: (−) negative, (+) 1-2 isolated Aβ depositions, (++) 3-4 Aβ depositions, and (+++) > 4 Aβ depositions. We also described different patterns of deposits and its intensity for Phos-Tau immunostaining : neurofibrillary tangles, neuropil threads and neuritic plaques (n.d: not determined; +: low; ++: intermediate; +++ high) (see Table 1). All preparations were examined by two independent pathologists.

### Materials

The following reagents and materials were used: anti-GAPDH (Calbiochem), anti-DPP6 (Sigma), anti-AKAP12, anti-CPNE6, anti-RIPX, anti-VILIP1 (Thermo Scientific). Electrophoresis reagents were purchased from Biorad and trypsin from Promega.

### Immunoblotting analysis

Equal amounts of protein (15 μg) were resolved in 12.5% SDS-PAGE gels. OB proteins derived from control, AD, PSP, LBD, FTLD, and mix AD VD were electrophoretically transferred onto nitrocellulose membranes for 45 min at 120 V. Membranes were probed with primary antibodies at 1:1000 dilution in 5% nonfat milk. After incubation with the appropriate horseradish peroxidase-conjugated secondary antibody (1:5000), the immunoreactivity was visualized by enhanced chemilumineiscence (Perkin Elmer). Equal loading of the gels was assessed by Ponceau staining and hybridization with a GAPDH specific antibody (Calbiochem). Results are expressed as an n-fold increase over the values of the control group in densitometric arbitrary units.

### Immunohistochemistry

For the immunohistochemical study, formalin-fixed sections (3-5 mm-thick) were mounted on slides and deparaffinized. Tissue sections were labelled with the following primary antibodies: anti-DPP6 (dilution 1/200), anti-AKAP12 (dilution 1/2500), anti-CPNE6 (dilution 1/500), anti-RIPX (dilution 1/500), anti-VILIP1 (dilution 1/100). The reaction product was visualized using an automated slide immunostainer (Leica Bond Max) with Bond Polymer Refine Detection (Leica Biosystems Newcastle Ltd).

### Sample preparation for proteomic analysis

OB specimens derived from control and AD cases (Table 1) were homogenized in lysis buffer containing 7 M urea, 2 M thiourea, 4% (w/v) CHAPS, 50 mM DTT. The homogenates were spinned down at 100.000 × g for 1 h at 15°C. Protein concentration was measured in the supernatants with the Bradford assay kit (Biorad). Prior to proteomic analysis, the individual OB samples were grouped into 8 independent pools containing ~ 200 μg of protein from 2-3 individual samples each one (see experimental design in Online Resource 1-Figure 1).

### Protein digestion and peptide iTRAQ labeling

A shotgun comparative proteomic analysis of OB proteomes using iTRAQ (isobaric Tags for Relative and Absolute Quantitation) was performed [45]. Protein extracts were precipitated with methanol/choloroform, and pellets dissolved in 7M urea, 2 M thiourea, 4% (v/v) CHAPS. Protein quantitation was performed with the Bradford assay kit (Bio-Rad). iTRAQ labeling of each pooled sample was performed according to the manufacturer's protocol (ABSciex). Briefly, equal amounts of OB proteins (80 μg) from each pool were reduced with 50 mM tris (2-carboxyethyl) phosphine (TCEP) at 60°C for 1 h. Cysteine residues were alkylated with 200 mM methylmethanethiosulfonate (MMTS) at room temperature for 15 min. Protein enzymatic cleavage was carried out with trypsin (Promega; 1:20, w/w) at 37°C for 16 h. Each tryptic digest was labelled according to the manufacturer's instructions with one isobaric amine-reactive tags as follows (see Online Resource 1-Figure 1): Tag113, control group A; Tag114, control group B; Tag115, Braak stage I-II group A; Tag116, Braak stage I-II group B; Tag117, Braak stage III-IV group A; Tag118, Braak stage III-IV group B; Tag119, Braak stage V-VI group A; Tag121, Braak stage V-VI group B. After 1h incubation, each set of labelled samples were independently pooled and evaporated until < 40 μl in a vacuum centrifuge.

### Liquid chromatography (LC)

To increase the proteome coverage, the peptide pool was injected to an Ettan LC system with a X-Terra RP18 precolumn (2.1 × 20mm) and a high pH stable X-Terra RP18 column (C18; 2.1 mm × 150mm; 3.5μm) (Waters) at a flow rate of 40 μl/min. Peptides were eluted with a mobile phase B of 5-65% linear gradient over 35 min (A, 5 mM ammonium bicarbonate in water at pH 9.8; B, 5 mM ammonium bicarbonate in acetonitrile at pH 9.8). 14 fractions were collected, evaporated under vacuum and reconstituted into 20 μl of 2% acetonitrile, 0.1% formic acid, 98% MilliQ-H_2_0 prior to mass spectrometric analysis.

### Mass spectrometry analysis

Peptides mixtures were separated by reverse phase chromatography using an Eksigent nanoLC ultra 2D pump fitted with a 75 μm ID column (Eksigent 0.075 × 150). Samples were first loaded for desalting and concentration into a 0.5 cm length 300 μm ID precolumn packed with the same chemistry as the separating column. Mobile phases were 100% water 0.1% formic acid (FA) (buffer A) and 100% Acetonitrile 0.1% FA (buffer B). Column gradient was developed in a 70 min two step gradient from 2% B to 30% B in 60 min and 30%B to 40% B in 10 min. Column was equilibrated in 95% B for 5 min and 2% B for 15 min. During all process, precolumn was in line with column and flow maintained all along the gradient at 300 nl/min. Eluting peptides from the column were analyzed using an AB Sciex 5600 Triple-TOF system. Information data acquisition was acquired upon a survey scan performed in a mass range from 350 m/z up to 1250 m/z in a scan time of 250 ms. Top 25 peaks were selected for fragmentation. Minimum accumulation time for MS/MS was set to 75 ms giving a total cycle time of 2.1 s. Product ions were scanned in a mass range from 100 m/z up to 1500 m/z and excluded for further fragmentation during 15 s.

### Data analysis

After MS/MS analysis, data files were processed using ProteinPilot™ 4.5 software from AB Sciex which uses the algorithm Paragon™ (v.4.0.0.0) [89] for database search and Progroup™ for data grouping and searched against Uniprot Human database. The search parameters allowed for cysteine modification by MMTS and biological modifications programmed in the algorithm (i.e. phosphorylations, amidations, semitryptic fragments, etc.). Reporter ion intensities were bias corrected for the overlapping isotope contributions from the iTRAQ tags according to the certiﬁcate of analysis provided by the reagent manufacturer (ABsciex). The peptide and protein selection criteria for relative quantitation were performed as previously described [90]. Several quantitative estimates provided for each protein by ProteinPilot were utilized: the fold change ratios of differential expression between labelled protein extracts; the p-value, representing the probability that the observed ratio is different than 1 by chance. A decoy database search strategy was also used to estimate the false discovery rate (FDR), defined as the percentage of decoy proteins identified against the total protein identification. The FDR was calculated by searching the spectra against the decoy database generated from the target database using a non-lineal fitting method [91] and displayed results were those reporting a 1% Global FDR or better. The results were then exported into Excel for manual data interpretation. Although relative quantification and statistical analysis were provided by the ProteinPilot software, an additional 1.3-fold change cutoff for all iTRAQ ratios (ratio ≤0.77 or ≥1.3) was selected to classify proteins as up- or down-regulated. Proteins with iTRAQ ratios below the low range (0.77) were considered to be underexpressed, whereas those above the high range (1.3) were considered to be overexpressed.

### Bioinformatic analysis

The proteomic information was analyzed using bioinformatic tools including DAVID (Database for Annotation, Visualization and Integrated Discovery) Bioinformatics Resources (v6.7), Panther, and Reactome tools [52, 92]. These programs detect and infer differentially activated/deactivated pathways as a result of AD phenotypes. The identification of specifically dysregulated regulatory/metabolic networks during the AD progression was analysed by STRING (Search Tool for the Retrieval of Interacting Genes) software (v.9.1) (http://stringdb.org/) [51]. This database includes interactions from published literature describing experimentally studied interactions, as well as those from genome analysis using several well-established methods based on domain fusion, phylogenetic profiling and gene neighbourhood concepts. A higher score was assigned when an association is supported by several types of evidence. To minimize false positives as well as false negatives, all interactions tagged as “low-confidence” (< 0.4) in STRING database have been eliminated from this study.

## SUPPLEMENTARY MATERIAL FIGURES AND TABLES















## Abbreviations

AD: Alzheimer's disease
AKAP12: A kinase anchor protein 12
DPP6: Dipeptidyl aminopeptidase-like protein 6
LC: Liquid chromatography
MS: Mass spectrometry
PPI: Protein-protein interaction
RIPX: RUFY3 protein
VILIP1: Visinin-like protein 1
PSP: Progressive supranuclear palsy
LBD: Lewy body disease
FTLD: Frontotemporal lobar degeneration
VD: vascular dementia
