# Supplementary material for: Olfactory bulb proteome dynamics during the progression of sporadic Alzheimer's disease: identification of common and distinct olfactory targets across Alzheimer-related co-pathologies
Source: Oncotarget. 2015 Oct 28;6(37):39437–56. doi: 10.18632/oncotarget.6254 (PMC4741837; doi:10.18632/oncotarget.6254)
Supplement: Supplementary file 1 [file oncotarget-06-39437-s001.pdf]

Olfactory bulb proteome dynamics during the progression of sporadic Alzheimer's disease: identification of common and distinct olfactory targets across Alzheimer-related co-pathologies

Supplementary Material

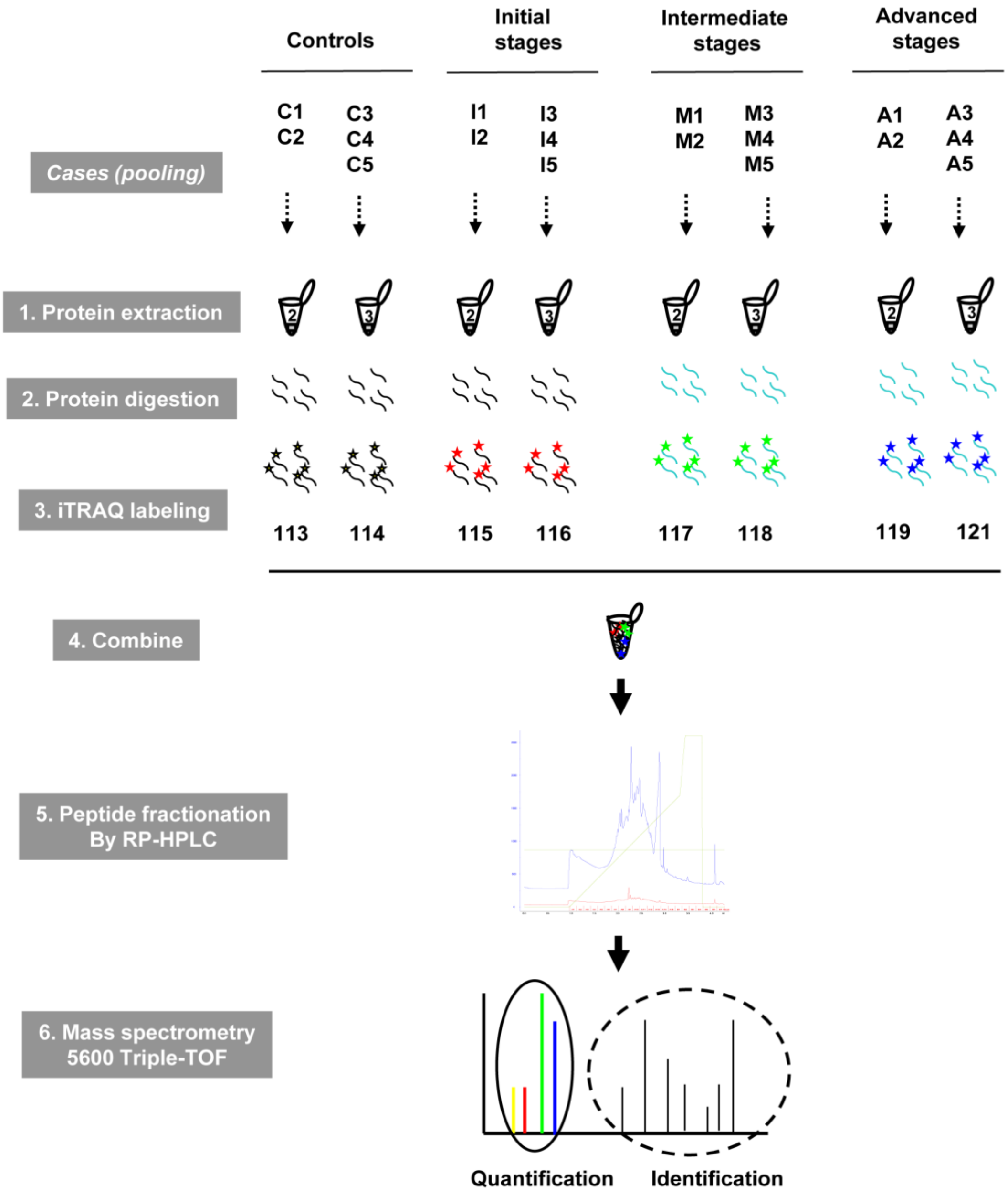

Supporting figure 1: Overview of the proteomic workflow used in this study

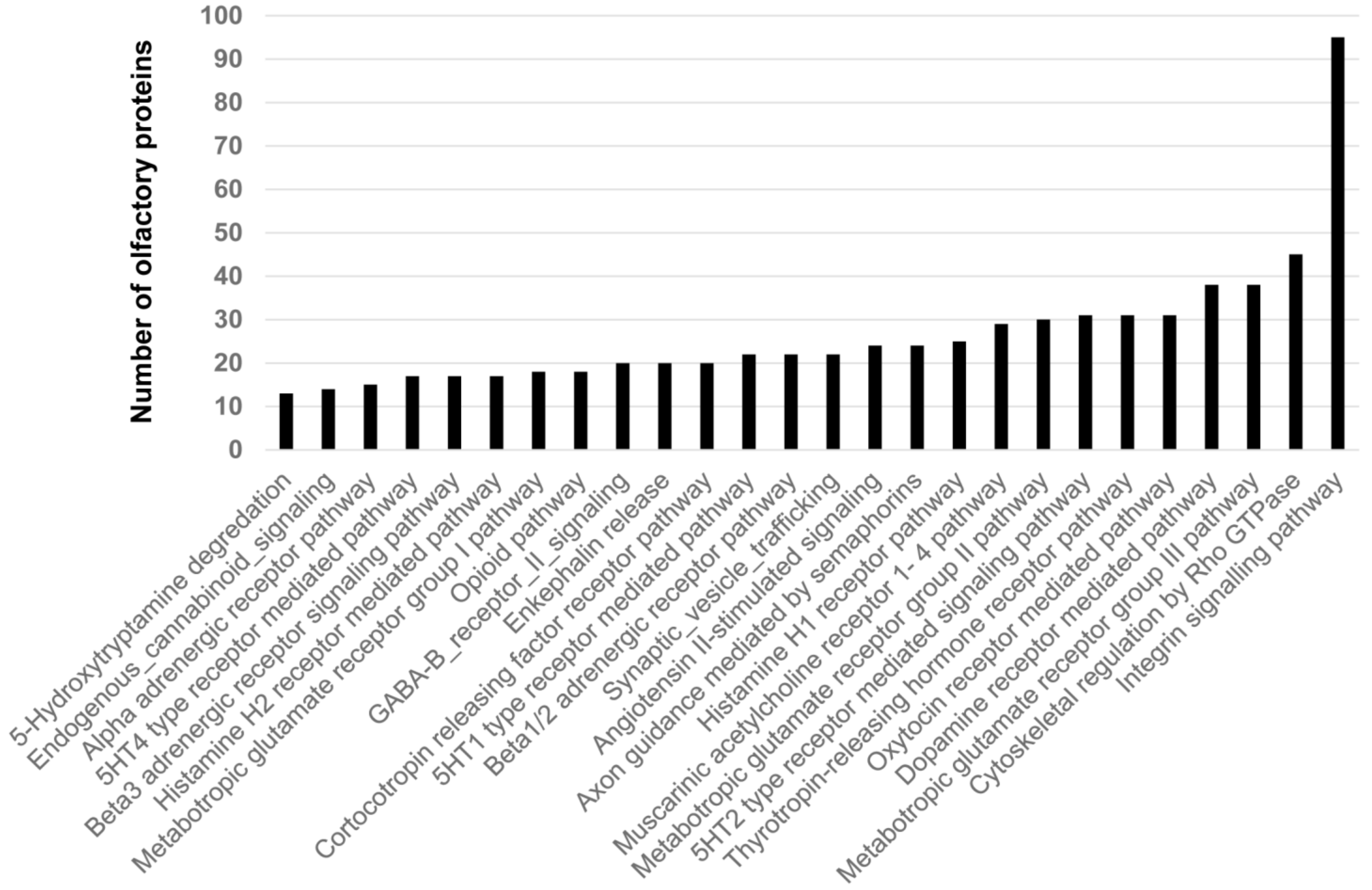

Supporting figure 2: A) Protein clusters significantly enriched in OB proteome dataset are shown (Panther tool: fold enrichment > 1.5, p-value <0.05). A complete characterization of each cluster is shown in supplementary table 3.

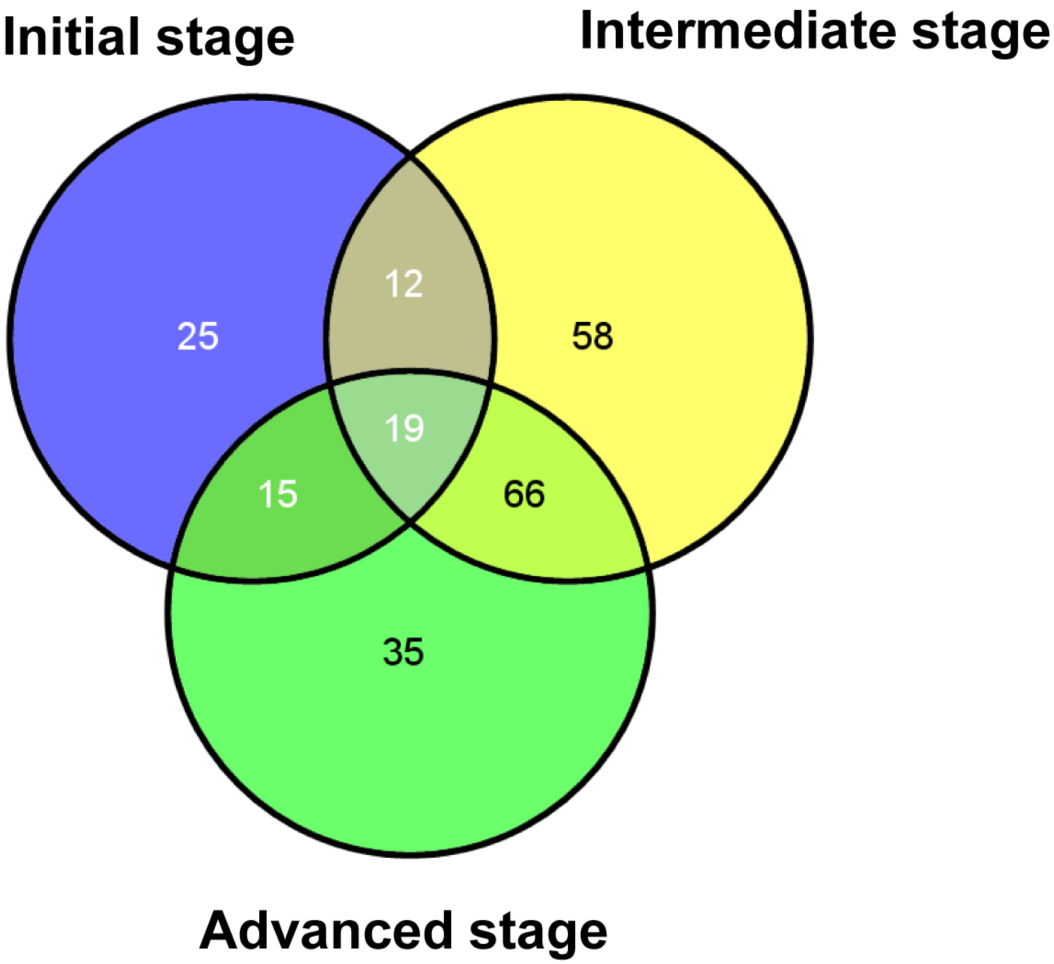

Supporting figure 3: Differential OB proteome that tend to be differentially expressed in each stage

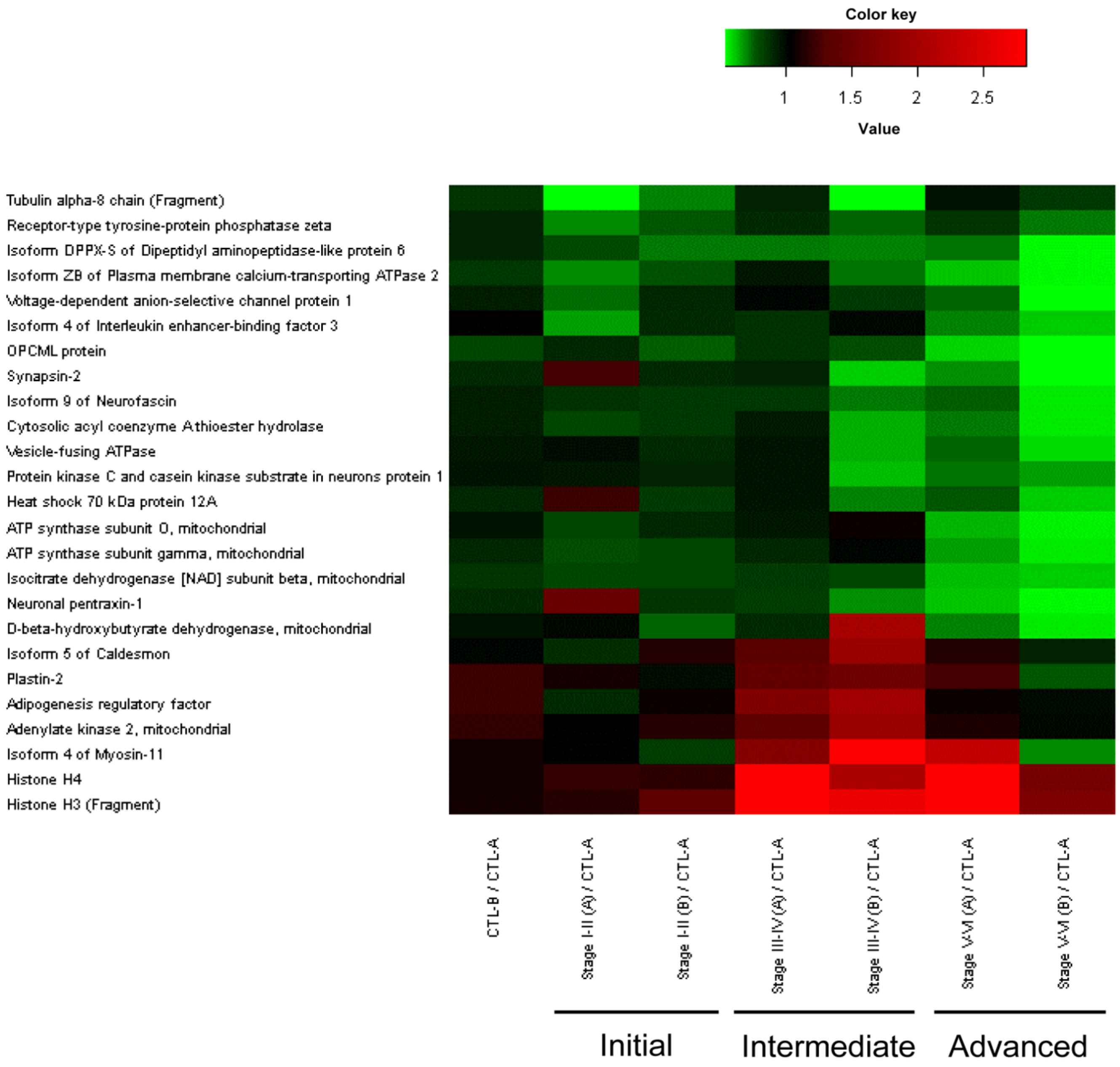

Supporting figure 4: Top proteins highly differentially expressed in specific stage compared to normally aged OB. Heat map shows the average change in protein expression among the four groups. Intensity values shown correspond to the ratio of the protein reporter ion intensity originating from control-B, initial stage (A;B), intermediate stage (A;B), and advanced stage (A;B) relative to control-A. Red-green color scale depicts protein expression levels (Red: overexpression and Green: underexpression).

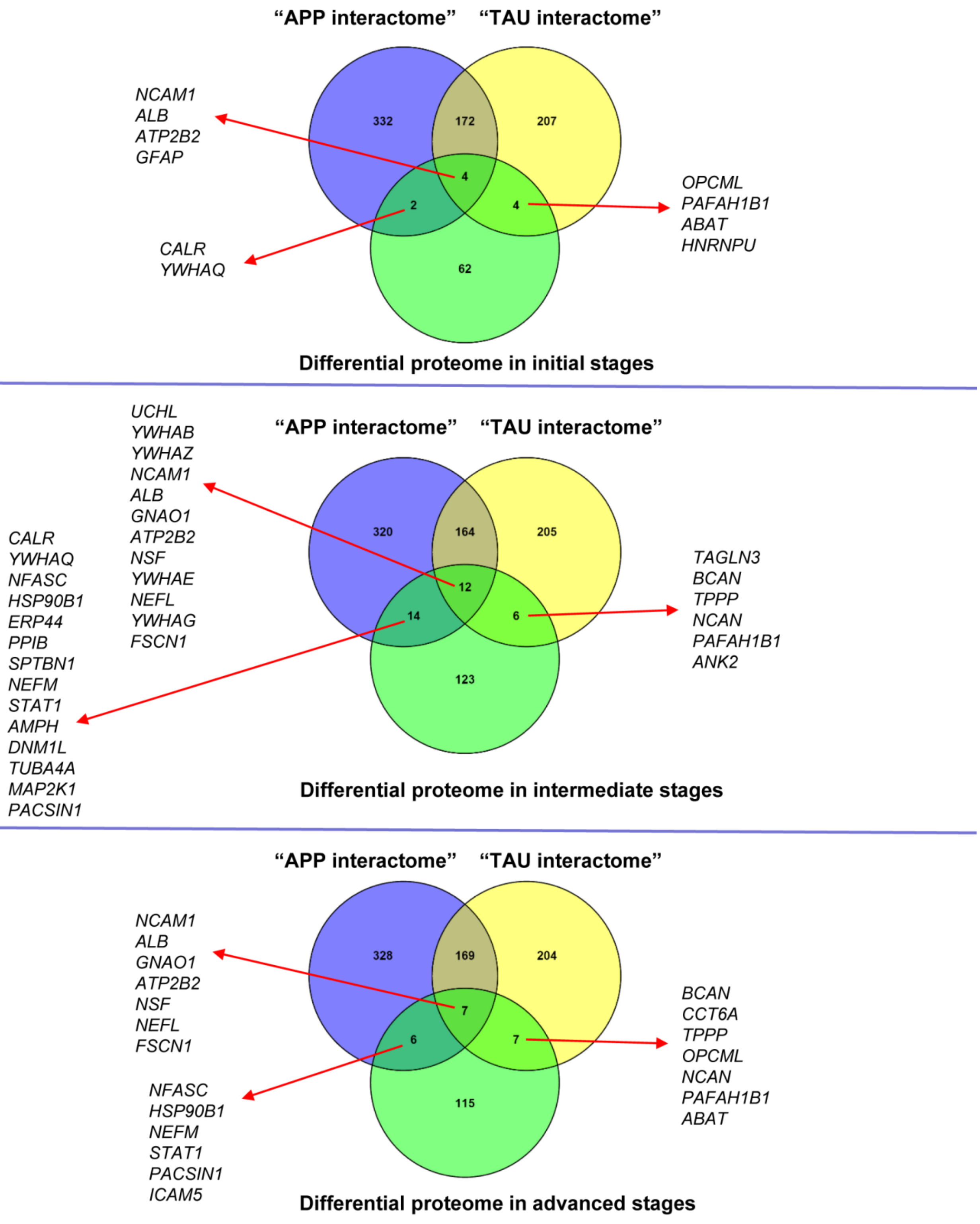

Supporting figure 5: Overlap between predicted APP and Tau interactomes and differential proteomes detected across Braak's stages. The predictions of interacting partners for human APP and Tau proteins have been obtained using FpClass tool (http://dcv.uhnres.utoronto.ca/FPCLASS/ppis/). Only binary interactions with a total score higher than 0.5 were considered. See Supplementary table 6 for more details).
